# Current Advances and Challenges in Radiomics of Brain Tumors

**DOI:** 10.3389/fonc.2021.732196

**Published:** 2021-10-14

**Authors:** Zhenjie Yi, Lifu Long, Yu Zeng, Zhixiong Liu

**Affiliations:** ^1^ Department of Neurosurgery, Xiangya Hospital, Central South University, Changsha, China; ^2^ XiangYa School of Medicine, Central South University, Changsha, China; ^3^ National Clinical Research Center for Geriatric Disorders, Xiangya Hospital, Central South University, Changsha, China

**Keywords:** radiomics, radiogenomics, glioma, pituitary tumor, brain metastases

## Abstract

Imaging diagnosis is crucial for early detection and monitoring of brain tumors. Radiomics enable the extraction of a large mass of quantitative features from complex clinical imaging arrays, and then transform them into high-dimensional data which can subsequently be mined to find their relevance with the tumor’s histological features, which reflect underlying genetic mutations and malignancy, along with grade, progression, therapeutic effect, or even overall survival (OS). Compared to traditional brain imaging, radiomics provides quantitative information linked to meaningful biologic characteristics and application of deep learning which sheds light on the full automation of imaging diagnosis. Recent studies have shown that radiomics’ application is broad in identifying primary tumor, differential diagnosis, grading, evaluation of mutation status and aggression, prediction of treatment response and recurrence in pituitary tumors, gliomas, and brain metastases. In this descriptive review, besides establishing a general understanding among protocols, results, and clinical significance of these studies, we further discuss the current limitations along with future development of radiomics.

## Introduction

Brain and other CNS tumors, including gliomas, pituitary tumors, and others such as brain metasteses, mainly occur in lung cancer and breast cancer patients. These tumors stand out for their high diversity and heterogeneity, along with dismal prognosis, ranking them among the top 10 causes of cancer deaths, accounting for a significant proportion of the deaths in men less than 40 years and women less than 20 years in the United States in 2018 (1), and it is estimated that they will cause 18,600 deaths in 2021.

**Figure 1:**
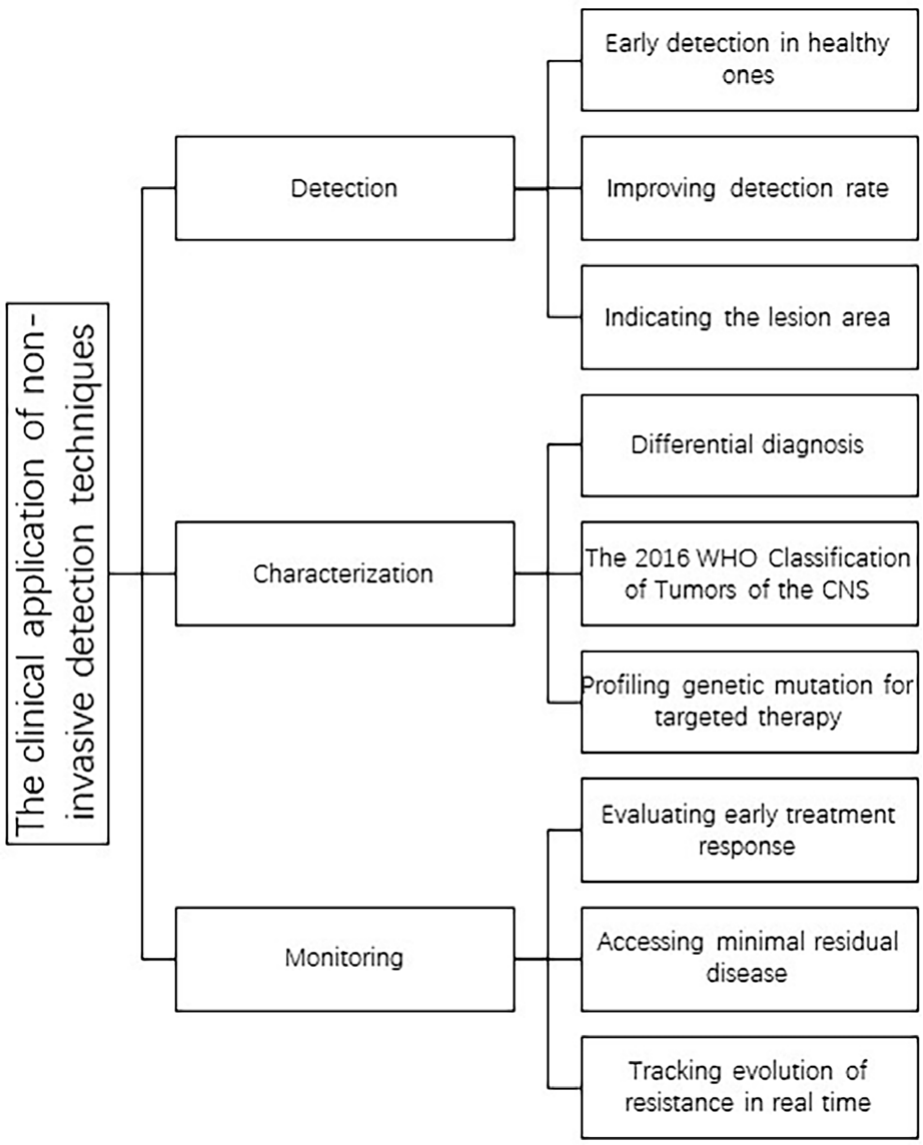
The clinical application of the noninvasive detection techniques.

Clinical radiology is a routinely performed examination for patients who are suspicious of brain or other CNS tumors; recently more and more sophisticated analytic methods have sprung up which supplement traditional imaging techniques. Based on the imaging techniques and incorporated with computer vision and machine learning (2), radiomics was born. Radiomics first appeared in Philippe’s review in 2012 (3), initially as an extended technique of computer-aided diagnosis and detection (CAD) systems. The term radiomics refers to the refining of a large mass of quantitative features from complex clinical imaging arrays, then transforming them into high-dimensional data which can subsequently be mined to find their relevance with the tumor’s histological features, which reflect underlying genetic mutations and malignancy, along with grade, progression, therapeutic effect, or even overall survival (OS) (4). Deep learning is a branch of machine learning, and machine learning is the necessary path to realize artificial intelligence (AI). The concept of deep learning originated from the study of neural networks that simulate the human brain. In recent years, we have seen a blossoming in AI development, with more intelligent algorithms such as deep learning bringing the possibility of realizing fully automatic image capturing and reading processes. Compared to traditional manual radiology practice requiring trained physicians to deal with large quantities of information, which is labor intensive, subjective, and qualitative, radiomics is able to use AI methods to provide automatic, objective, and quantitative data with high efficiency. In this review, we focus on radiomics and extended imaging techniques. The general clinical applications of these noninvasive methods are shown (**Figure 1**).

### Clinical Application

Early accurate diagnosis and classification are crucial in prolonging the patient’s survival time. The application of radiomics has been initiated in clinical oncology early diagnosis, since its ability to analyze the combination of numerous quantitative features provides the possibility to unravel the underlying pathophysiology that is hard to be perceived by radiologists’ eyes and avoid subjective misreading. The general workflow of radiomics involves several discrete steps: imaging, segmentation, feature extraction, feature selection, machine learning, and validation (5). Segmentation is using a series of algorithms to delineate regions of interest (ROIs), which refers to the tumor and its surrounding abnormality from other tissue, and then further subdividing the legion by its intra-heterogeneity to facilitate the next steps. Accurate segmentation is a key step from image processing to quantitative analysis, acting as a prerequisite for the subsequent diagnostic tasks like defining the location, extent of radiation, and tumor feature extraction, and is the most challenging step due to high heterogeneity and irregularity of brain tumors. Feature extraction refers to the use of existing features to calculate a feature set with a higher degree of abstraction, and also refers to an algorithm for calculating a certain feature. Then the extracted features will go through feature selection, which aims at reducing dimensionality and the difficulty of learning tasks to improve the efficiency of the model. The top features will be integrated with clinical results and/or pathological results, together as input into machine learning methods to build prediction or classification models.

The main clinical tasks of radiomics lie in three parts: detection, characterization, and monitoring. By the help of computer-aided detection (CADe), the suspicious area of the image can be highlighted and some features indicating early cancer lesions can be detected, which reduce observational oversights and improve the speed of interpretation (6, 7). Most radiomics models are served for characterization, including diagnostic tasks (differential diagnosis, malignancy, WHO CNS classification, specific genetic mutation status, and treatment effect) and predictive/prognostic tasks (treatment effect, OS/PFS, complication, and tumor recurrence). Monitoring is also significant within clinical practice in evaluating the progression of tumor and the effect of treatment. Traditional protocols to assess the tumor progression, recommended by institutions like Response Evaluation Criteria in Solid Tumors (RECIST) and WHO, are usually defined by the size of tumor (8), which omits much geometry and material information detected by advanced radiological instruments and also oversimplifies the indicators on tumor burden. The emergence of AI-monitoring may help radiologists to establish more sophisticated quantitative protocols towards tumor burden evaluation.

MRI acts as a key part and is usually the first choice in radiological diagnosis of brain tumors. First, MRI has an outstanding contrast capacity for the detection of brain tissues. Second, MRI has many different sequences respectively sensitive to different physiology parameters, such as blood flow and edema in surroundings, which indicate the tumor’s microenvironment. Third, MRI can be implemented throughout the treatment noninvasively and assesses the progression and effect. Besides this anatomical imaging, the multimodal MRI with emerging sequences and technical developments like PET using either amino acid, choline, or fluorodeoxyglucose, as well as fusion PET/CT and PET/MRI scanners, provides a mass of functional neuroradiological information towards tumor penetration boundaries and heterogeneity in brain tumor patients (9, 10).

### Radiogenomics

In 2016, WHO published a new guideline on classifying CNS tumors; the molecular markers are especially spotlighted to describe brain tumor entities histology features for the first time, which leads to more precise tumor cataloging (11). Furthermore, target therapies and treatment strategies for malignant brain tumor patients are also predominantly dependent on specific molecular markers, emphasizing the importance of precision oncology. With the advance of big data and bioinformatics, it is possible to detect the correlations between gene expression and radiomics features, which is known as radiogenomics. Radiogenomics is based on a common hypothesis that the dissimilarities in phenotypes of ROIs can be attributed to gene-expression patterns (12). Panth et al. further proved that genetic changes that lead to phenotypic consequences can be reflected in variations of radiomics features (13). The main tasks of radiogenomics are to investigate the correlation between germline genotypic variance and the large clinical post-radiotherapy variability (14), as well as the correlation between specific imaging features and the inherent cellular pathophysiology (15).

Radiogenomics is analogous to the combination of radiology and genomics, but people should be aware that a sole radiomics analysis without biopsy genomics confirmation is not robust enough for definitive assessment of gene expression or other contents in ROIs. On the one hand, radiomics or radiogenomics only reveals the correlation between features and genetic alterations, not the causes. On the other hand, not all of the phenotypic differences are induced only by genetic alteration, but also epigenetic changes and other factors. Thus, they actually incorporate with histopathologic examinations or sequencing, which provides confirmatory information to improve clinical decision making. Radiogenomics holds great potential for an expanding translational technology, mainly due to three characteristics:

First of all, while genomic sequencing usually uses biopsy samples from one representative part of the tumor, the radiomics data is derived from the entire tumor lesion, so the outcome can capture radiomics features on the whole. As a result, radiogenomics supplements genomic sequencing with intratumoral heterogeneity and even intertumoral heterogeneity. Several recent studies have exhibited the role of radiogenomics in identifying regional genetic heterogeneity in malignant tumor with broad genetic diversity that led to treatment resistance such as glioblastoma (GBM) (16–18).

The following advantage lies in that radiogenomics are easy, rapid, noninvasive, and dynamic, as well as cost-effective. As imaging becomes routine for patients who are suspicious of brain tumor and the estimated error rate of cancer histopathology can be as high as 23% (19–22), quantitative imaging provides additional information to avoid observer variability and indicates actual biopsy sites. For those who have contraindications of biopsy, radiomics or radiogenomics is expected to serve as a secondary substitution to guide individualized medicine. As far as costs are concerned, radiomics are usually low cost compared to biopsy; it costs around 2000 dollars for a brain biopsy in China, but less than half of that for radiomics. In addition, brain biopsy is an invasive procedure that includes risks like bleeding, seizure, infection, and even paralysis or death for key lesions like basal area and brain stem. Consequently, radiomics outweighs biopsy in costs and risks. Since radiomics or radiogenomics is easy and rapid, they enable the monitoring of the change of gene expression in the tumor’s different regions, which may potentially indicate the causes of gene mutation.

Finally, there are single features strongly related to genes, and a cluster of features not significantly correlative to genes, but that have the potential to provide the information with some sort of combination, which has made progress in predicting cancer immunotherapy. By combining CT radiomics features and genomic data based on CD8B, Sun et al. developed a novel radiomics-based biomarker to predict CD8 cell count and clinical outcomes of patients’ response to anti-PD-1 or anti-PD-L1, when validated by further prospective randomized trials (23). In an AI-based radiomics study by Trebeschi et al., the biomarker mainly regarding tumor proliferation could predict anti-PD-1 therapy response with an AUC of up to 0.76 for both advanced melanoma and non-small-cell lung cancer (NSCLC) patients (24). It is also promising to see more robust radiomics-based biomarkers on targeted therapy in the future, such as antiangiogenic treatment with bevacizumab (25). To improve the resolution and confidence of features’ subsets related to gene modification or expression, big data from multiple centers should be collected and integrated.

## Pituitary Tumor

Pituitary adenomas (PAs) are among the leading types of brain tumors, and the foremost frequent lesion in sellar area. Usually, the hormone hypersecretion is assessed by immunocytochemical or hormone assays to distinguish secretory tumor from non-secretory ones. And the evaluation of tumor mass, such as accurate location and volume, is based on diagnostic imaging and visual field examination. Accurate clinical diagnosis derived from tumor characteristics helps individualized treatment. Radiomics will likely never replace histopathology or hormonal diagnosis for adenomas. However, separately, AI algorithms may replace the work performed by pathologists in interpreting microscopic analyses. Recent progress in pituitary tumors, gliomas, and brain metastases are arranged and summarized in **Tables 1**, **2**.

**Table 1:** Radiomic.

Study	No. of patients involved (Training/Test)	General purpose	CT or MRI contrast(s)	Segmentation method	Nomber of features (extraction/selection)	Feature selection method	Classification method	Performance (Training/Test)
Yae Won Park et al. (30)	141/36	To predict the DA response in prolactinoma patients	T2WI	^^3D Slicer software	107/n.a.	/	soft voting (RF, light gradient boosting machine, extra-trees, quadratic discrimination analysis, and linear discrimination analysis)	#0.81/0.81 (AUC)
Yang Zhang et al. (40)	50/n.a.	To predict progression or reccurence in NFPAs	CE-T1WI and T2WI	^^^fuzzy c-mean (FCM) clustering algorithm	107/3	SVM	SVM	***0.78/n.a.(AUC)
Andrei Mouraviev et al. (117)	87/n.a.	To predict local recurrence following SRS	CE-T1WI and FLAIR	^^Elastix software	440/12	Random forest feature importance (RFI)	RF	#0.793/n.a. (AUC)
Kniep, H et al.(113)	189/n.a.	To predict tumor type in different BM with unknown primary lesion	CE-T1WI, T1WI and FLAIR	^^Analyze 11.0 Software	1423/20	Decision tree	RF	*0.64 (NSCLC)-0.82 (MM)/n.a.(AUC)
Peng, LK et al. (116)	66/n.a.	To predict diagnosing treatment effect after stereotactic radiosurgery	CE-T1WI and FLAIR	^^a multiparametric deep learning (MPDL) network	51/5	IsoSVM	IsoSVM	**0.81/n.a.(AUC)
Ji Eun Park et al. (73)	85/35	To predict core signaling pathways in IDH wild-type GBM	T1WI, T2WI, DWI, FLAIR, CE-T1WI, and DSC	^^MITK software	71/5(RTK), 17/5(P53), 35/5(retinoblastoma)	t-test, LASSO, RF	Logistic regression	(3-fold CV)0.87/0.88(RTK), 0.80/0.76(p53), 0.84/0.81(retinoblastoma pathway) (AUC)
Pascal O. Zinn et al. (65)	46/47(GBM), 40/n.a.(mice)	To establish causality between POSTN status and MRI-extracted radiomic-features in GBM	FLAIR and CE-T1WI	^^3D Slicer software	2480/48(GBM),17(mice)	LASSO	Binary logistic regression	**76.56%/n.a.(GBM), 92.26%/n.a.(mice)(AUC)
Chia-Feng Lu et al. (46)	214/70	To stratify the molecular subtypes of gliomas	CE-T1WI, FLAIR, T2WI, and DWI	^^n.a.	39212/(20-1960)	Two-sample t-test with pooled variance estimate	SVM	*87.7%-96.1%/80.0%-91.7%(accuracy)
Robin Gutsche et al. (47)	50/n.a.	To evaluate the repeatability of feature-based FET PET radiomics and investigate IDH genotype on feature repeatability	FET PET	^^^TBR≥1.6	1302/297	intraclass correlation coefficient	n.a.	n.a.
Yoon Seong Choi et al. (48)	727/439(129 internal and 310 external)	To predict the IDH status of gliomas	CE-T1WI, T2WI and FLAIR	^^^CNN	24/20	CNN	CNN	***0.96/0.94(internal), 0.86(external)(AUC)
Anna Luisa Di Stefano et al. (67)	66/78	To characterize the clinical, radiological, and molecular profile of F3T3 positive diffuse gliomas	T1WI, CE-T1WI and FLAIR	^ITK-SNAP software	2616/25	Cox proportional hazards models (OS)	RF(F3T3)	*F3T3: 0.87/0.745(AUC)
Xiaorui Su et al. (71)	75/25	To predict H3 K27M mutation status in midline gliomas.	FLAIR	^ITK-SNAP software	99/10	TPOT	TPOT	*** 0.788~0.867/0.60~ 0.84(accuracy)
Zev A. Binder et al. (59)	260/n.a.	To investigate the negative survival impact of EGFRA289D/T/V	T1WI, CE-T1WI, T2WI, FLAIR, DTI, and DSC	^^GLISTRboost	2104/299/17	SVM/radiographic interpretability	n.a.	n.a.
Jingwei Wei et al. (50)	74/31	To predict MGMT methylation status in astrocytoma	CE-T1WI, FLAIR and DWI	^ITK-SNAP software	3051/13	The minimum redundancy maximum relevance (mRMR)	logistic regression	#0.925/0.902(AUC)
Yiming Li et al. (51)	63/123(32 internal and 91 external)	To predict ATRX mutation in LGGs	T2WI	^MRIcro software	431/9	LASSO	SVM	#0.94/0.925(internal) and 0.725(external)(AUC)
Johannes Haubold et al. (52)	28/14	To predict tumor grading and mutational status of patients with cerebral gliomas	CE-T1WI, ADC, and 3D-FLAIR (SPACE), FET PET, and SWI, The water-content-based M0 map (MRF M0) , T1FLAIR, DWI	^^3D Slicer software	19284/32(1p19q codeletion), 64(IDH1), 8(ATRX),16(MGMT)	f score (ANOVA), chi-square, LCSI and randomized logistics regression (RandLR)	RF and SVM	*WHOI-IV:0.818/n.a.(AUC); Differentiation of LGG and HGG:0.85/n.a.(AUC); 1p19q codeletion: 0.9784/n.a.(AUC); IDH1:0.88/n.a.(AUC); ATRX:0.851/n.a.(AUC); MGMT:0.757/n.a.(AUC)
Luyuan Zhang et al. (57)	96/24	To identify the value of CIC mutations in gliomas	T1WI, T2WI, FLAIR and CE-T1WI	^FSL image viewer	6676/11	LASSO	logistic regression	*0.985/n.a.(AUC)
Changliang Su et al. (53)	220/n.a.	To differentiate among glioma subtypes and predict tumour proliferation	T2WI fast-echo images (T2FSE), T1WI, FLAIR, CE-T1WI, DWI, PWI and CBF	^^ImageJ	431/25	univariate analysis	logistic regression	#0.936/n.a.(AUC)
Yiming Li et al. (76)	69/40	To predict PTEN mutation status in GBM	T1WI, T2WI and CE-T1WI	^MRIcro software	862/6	The minimum redundancy maximum relevance (mRMR)	SVM	#0.925/0.787(AUC)

^ manual segmentation; ^^ semi-automatic segmentation; ^^^ full-automatic segmentation; * 5-fold cross-validation; ** leave-one out cross-validation; *** 10-fold cross-validation; # cross validation not available;

AUC, area under the receiver operating characteristic curve; GBM, glioblastoma; BM, brain metastases; PsP, pseudoprogression; CCC, concordance correlation coefficient; IDH, isocitrate dehydrogenase; PCNSL, primary central nervous system lymphoma; TBR, tumor-to-brain ratio; CNN, convolutional neural network; LASSO, least absolute shrinkage and selection operator; MGMT, O6-methylguanineDNA-methyltransferase; EGFR, epidermal growth factor receptor; TRC, treatment-related changes; RFE, recursive feature elimination; RF, random forest; SAM, significance analysis of microarrays; SVM, support vector machine; n.a., not available; PCA, principal component analysis;

MRI, magnetic resonance imaging; mRMR, minimum redundancy maximum relevance algorithm; MRS, magnetic resonance spectroscopy; DTI, diffusion tensor imaging; DWI, diffusion-weighted imaging; FLAIR, fluid-attenuated inversion recovery; PWI, perfusion-weighted imaging; T1, T1-weighted MRI; CE-T1WI, contrast-enhanced T1-weighted MRI; T2, T2-weighted MRI; DSC, dynamic susceptibility contrast; CBV, cerebral blood volume.

**Table 2:** Radiomic.

Study	No. of patients involved (Training/Test)	General purpose	CT or MRI contrast(s)	Segmentation method	Nomber of features (extraction/selection)	Feature selection method	Classification method	Performance (Training/Test)
Daesung Kang et al. (82)	112/42	To identify atypical PCNSL mimicking GBM	T1WI, T2WI, FLAIR, DWI, CE-T1WI and DSC	^^MITK software	1618/15(ADC), 40(CE-T1WI)	RFE(ADC); relief (CE-T1W1)	RF(ADC); LDA(CE-T1WI)	***0.983(AUC)/0.984(internal AUC)0.944(external AUC)
Guoqing Wu et al. (83)	67/35	To differentiate PCNSL and GBM and for IDH1 mutation estimation	T2WI and CE-T1WI	^^^CNN	968/49	Sparse representation	Collaborative sparse representation	**98.51%/94.51%(accuracy)
Zenghui Qian et al. (81)	227/185	To differentiate GBM from BM preoperatively	T1WI, T2WI and CE-T1WI	^MRIcro software	1303/24	LASSO	SVM	*0.945/0.90(AUC)
Jung Youn Kim et al. (86)	61/57 (23 internal and 34 external)	To differentiate PsP from early tumor progression in patients with GBM	CE-T1WI, FLAIR, ADC and CBV maps	^^MITK software	6472/12	LASSO logistic regression model	Student’s t-test and the chi-square test	***0.9(AUC)/0.96(internal AUC), 0.85(external AUC)
Katrin Aslan et al. (91)	148/n.a.(mice)	To predict treatment response and PsP in ICB-treated mice	T2WI and CE-T1WI	^Osirix or ITKsnap software	423/423	/	Gradient boosting	*82.7%/n.a. (accuracy)
Nabil Elshafeey et al. (84)	98/7	To discriminate PsP from progression in GBM	FLAIR, T1WI and CE-T1WI	^^3D Slicer software	620/60	Maximum Relevance Minimum Redundancy (MRMR)	SVM	**89%/n.a.(AUC)
Jinhua Cai et al. (90)	77/72 (41 internal and 31 external)	To predict the response to bevacizumab in patients with brain necrosis after radiotherapy	FLAIR	^^3D Slicer software	1301/18	LASSO	multivariate logistic	#0.916/0.912 (internal) and 0.827 (external)(AUC)
Philipp Lohmann et al. (85)	72/30	To differentiate PsP in glioma patients post-chemoradiation	FET PET	^^^TBR=1.4, 1,6, 1.8	944/4	RFE	TPOT	*0.74/0.74 (AUC)
Kai Wang et al. (87)	112/48	To discriminate tumor recurrence from radiation necrosis in glioma	CE-T1WI, FLAIR, 18F-FDG and 11C-MET PET	^ITK-SNAP software	396/15	LASSO	Multivariable logistic regression analysis	***0.988/0.914(AUC)
Zi-Qi Pan et al. (94)	82/70 (40 internal and 30 external)	To predict the response of individual GBM patients to radiotherapy	T1WI, CE-T1WI, T2WI, and FLAIR	^^GLISTR software	28496/8	Boruta algorithm	RF	***0.764/0.758 (external)(C-index)
Xing Liu et al. (103)	216/84	To predict the PFS in LGGs and investigate the corresponding genetic background	T2WI	^MRIcro software	431/9	Univariate Cox regression	LASSO Cox regression	***0.684/0.823(C-index)
Sara Dastmalchian et al. (95)	31/n.a.	To differentiate between intra-axial adult brain tumors and to predict survival in the GBM cohort	T1WI, T2WI, FLAIR and CE-T1WI	^Magnetic resonance fingerprinting	39/20	Spearman’s rank correlation coefficient test	Wilcoxon rank sum	#Peripheric white matter regions in GBM and LGGs: 0.869/n.a.(AUC), Solid tumor regions in LGGs and metastases: 0.952/n.a. (AUC)
Zenghui Qian et al. (102)	85/148	To identify OS in patients with LGGs	T2WI	^MRIcro	55/6	univariate Cox regression	multivariate Cox regression	#0.92/0.70(C-index)
László Papp et al. (97)	42/28	To estimate survival for glioma	11C-MET PET	^^Hybrid 3D	112/56	Genetic algorithm and Nelder–Mead method	geometric probability covering algorithms	Monte Carlo cross-validation, n.a./0.9(M36IEP AUC)
Sohi Bae et al. (96)	163/54	To predict survival in patients with GBM	T1WI, T2WI, FLAIR, 3D CE-T1WI and DTI	^^signal intensity thresholding, region growing, and edge detection	796/18	Random survival forest	Random survival forest	***Overall survival: n.a./0.652, Progression-free survival: n.a./ 0.590(iAUC)
Niha Beig et al. (98)	83/113 (male), 47/70 (female)	To develop sexually dimorphic radiomic risk score (RRS) models that are prognostic of OS	CE-T1WI, T2WI and FLAIR	^^CapTK	105/8 (male), 287/6 (female)	LASSO	Cox regression	#0.73/0.88(C-index, male), 0.73/0.69(C-index, female)
Maikel Verduin et al. (99)	142/46	To established prognostic clinical features, predict IDH-mutation, MGMT-methylation and EGFR amplification develop a prognostic model for OS in GBM	CE-T1WI and T2WI	^Osirix Lite and MiM software	1197/5	XGBoost	Cox-regression	***0.72/0.73(C-index)
Philipp Kickingereder et al. (100)	120/61	To stratify patients with GBM	CE-T1WI, T2WI and FLAIR	^^ITK-SNAP	1043/386	CCC	Lasso-penalized Cox model (Coxnet algorithm)	***0.103(all layers, OS IBS); 0.089(all layers, PFS IBS)

^ manual segmentation; ^^ semi-automatic segmentation; ^^^ full-automatic segmentation; * 5-fold cross-validation; ** leave-one out cross-validation; *** 10-fold cross-validation; # cross validation not available;

AUC, area under the receiver operating characteristic curve; GBM, glioblastoma; BM, brain metastases; PsP, pseudoprogression; CCC, concordance correlation coefficient; IDH, isocitrate dehydrogenase; PCNSL, primary central nervous system lymphoma; TBR, tumor-to-brain ratio; CNN, convolutional neural network; LASSO, least absolute shrinkage and selection operator; MGMT, O6-methylguanineDNA-methyltransferase; EGFR, epidermal growth factor receptor; TRC, treatment-related changes; RFE, recursive feature elimination; RF, random forest; SAM, significance analysis of microarrays; SVM, support vector machine; n.a., not available; PCA, principal component analysis;

MRI, magnetic resonance imaging; mRMR, minimum redundancy maximum relevance algorithm; MRS, magnetic resonance spectroscopy; DTI, diffusion tensor imaging; DWI, diffusion-weighted imaging; FLAIR, fluid-attenuated inversion recovery; PWI, perfusion-weighted imaging; T1, T1-weighted MRI; CE-T1WI, contrast-enhanced T1-weighted MRI; T2, T2-weighted MRI; DSC, dynamic susceptibility contrast; CBV, cerebral blood volume.

### Evaluation of Aggression

Ki-67 is only expressed in the nucleus of proliferating cells and the Ki-67 labeling index is considered to be an ideal indicator for detecting cell proliferation activity. PAs may exhibit clinically invasive or aggressive behavior, accompanied by postoperative recurrence and resistance to multidimensional therapy, which is usually evaluated by Ki-67 labeling index through immunohistochemistry. Ugga et al. collected 89 patients with available Ki-67 data and performed k-nearest neighbors (k-NN) to build a radiomics classifier using 12/1128 quantitative radiomics features to evaluate the Ki-67 labeling index based on preoperative T2WI MRI, which is effective and accurate (accuracy 91.67%) (63).

Knosp grade describes the degree of PA invasion to bilateral cavernous sinuses (CS). Niu et al. predicted CS invasion in 194 PA patients (training cohort: 97; test cohort: 97) graded 2-3 with Knosp pre-operatively by radiomics approaches deriving from contrast-enhanced T1 (CE-T1) and T2WI MRI (64). They applied LASSO to select three important features and establish a classifier using support vector machine (SVM), which yielded decent AUC values (training cohort: 0.852, test cohorts: 0.826).

### Grading

The regulation of adenohypophyseal cell differentiation and hormone secretion are operated by a series of transcription factors, including Tpit, Pit-1, and SF-1. Peng et al. involved 235 patients with pituitary adenoma (PA), and 18 quantitative imaging features were verified as significant to train SVM, k-NN, and Naïve Bayes (NBs) models to classify the transcription factor types of PAs. Among the three models, the SVM model showed the best performance (AUC 0.9549) whereas the K-NN (AUC 0.9266) and NBs (AUC 0.9324) models displayed lower performance and they found better performance in T2-weighted than Tl-weighted and CE-T1 (65).

Zhang et al. worked on differential diagnosis of non-functioning pituitary adenomas (NFPAs) subtypes from other subtypes preoperatively (66). They enrolled 75 patients as the training cohort and 37 patients as the test cohort, and derived complete T1-WI and CE-T1 MRI. The top three T1-WI imaging features, rather than CE-T1 imaging features, were ultimately selected using mRMR to fit a Radial Basis Function (RBF)-SVM predictive signature. A nomogram incorporated clinical characteristics and the radiomics signature corresponding to the best predictive model for individual prediction. Finally, the calibration of the nomogram was presented with a concordance index (CI) (training cohort: 0.854, test cohort: 0.857).

### Prediction of Treatment Response

Prolactinoma is the most common secretory PA, with prime treatment being dopamine agonists (DA) such as bromocriptine. To assess prolactinoma patients’ response to DA before initiating the treatment plan, Park et al. investigated a total of 177 prolactinoma patients’ coronal T2-weighed MRIs and set up a radiomics predictor with an AUC of 0.81 in both training and test cohorts (26).

Acromegaly is a severe complication that leads to poor prognosis most frequently caused by somatotroph PAs that secrete excessive growth hormone (GH). For those who are not suitable for surgery or have severe symptoms, somatostatin receptor ligand (SRL) treatment is usually applied to reduce the volume of mass preoperatively (67–69). To predict the treatment response of SRL ahead of surgical resection, radiomics features from MRI are selected to predict the histological granulation pattern. Park et al. set up a radiomics prediction model based on 69 GH-secreting PA patients and the model showed an AUC of 73.7% (70).Fan et al. proposed a radiomics prediction model of invasive functional pituitary adenoma (IFPA) working on the treatment responses before surgery (71). The prediction model was incorporated with a radiomics signature based on seven selected features derived from MRI and Knosp grade of each IFPA patient. The discrimination abilities and calibration of this yielded good performance, with AUCs (training cohort: 0.832, test cohort: 0.811).

### Prediction of Recurrence

Early progression/recurrence (P/R) is a poor prognostic factor of macro NFPAs that is reported in 25–55% patients after surgical resection (72–75). Zhang et al. established a SVM radiomics model based on three selected features from 50 patients’ 214 preoperative and postoperative follow-ups MRI features extracted from CE-T1 and T2WI yielded an overall accuracy of 82% and AUC of 0.78 discriminating P/R NFPAs from non-P/R ones using the original mask tumor ROI (27). They also calculated SVM scores for each patient and found that higher scores may correlate with shorter PFS. Regarding SVM score for differentiation of P/R, the optimal cut-off value was calculated which means patients with SVM scores higher than 0.537 tended to exhibit shorter PSF and the corresponding AUC (0.87) were obtained.

Machado et al. applied 3D CE-T1 MRI of patients preoperatively and segmented two and three-dimensional regions. They evaluated the 255 extracted radiomics features of 27 patients with NFPA and selected six features for two-dimensional machine learning radiomics models and 13 for three-dimensional models (76). The results showed 3D-feature based models have superior discrimination ability to classify NFPAs recurrent and stable lesions than 2D-feature based models, with their accuracies of up to 96.3% compared to accuracies of 92.6% for models solely based on 2D features.

## Gliomas

Gliomas account for the first leading primary brain and other CNS tumor in adults (25.1%) (77), making up approximately 80.8% among primary malignant brain and other CNS tumors with certain diagnoses (11).As the majority of gliomas (57.7%), GBM accounts for the foremost and lethal primary malignant brain tumor in neurosurgery, whose overall incidence rate is estimated 0.0032% in American adults (78). Though multimodal treatment protocols, including maximal safe surgical resection and adjuvant radiation therapy with concurrent chemotherapy mainly temozolomide (TMZ), are standardly given to GBM patients, they still suffer a crushingly adverse prognosis with 14.6 months of median OS (79).

### Evaluation of Mutation Status

Isocitrate dehydrogenase (IDH) mutations are recognized as a good prognostic factor in early clinical intervention and can be integrated into routine clinical practice such as pathological examination *via* radiomics analysis, immunohistochemistry, flow cytometry, standard PCR, and/or sequencing techniques (80). All IDH-mutant diffuse astrocytic tumors are classified as a single type of tumor (astrocytoma, IDH mutation, grade 2/3/4) and all GBM only included IDH wild type tumors in the 2021 WHO CNS 5. The 1p19q co-deleted tumors are exclusively oligodendrogliomas. And most oligodendrogliomas with 1p/19q co-deleted, which indicates poor prognosis, are accompanied by IDH1 and IDH2 mutation. In 2016 WHO CNS 4, the guideline of gliomas classification incorporated five molecular subtypes of diffuse gliomas based on IDH mutation and 1p/19q codeletion status.

Lu et al. built a multilevel quantitative imaging model based on CE-T1 image, T2 FLAIR, T2WI, DWI, and ADC to recognize IDH and 1p/19q genotypes of glioma and further classification of five molecular types (33). The training cohort involved 214 patients and an additional independent cohort involved 70 patients for external test. The IDH and 1p/19q classifier using SVM models was established in the training cohort, yielding areas under receiver operating characteristic (ROC) curves ranging from 0.922 to 0.975, and accuracies ranging from 87.7% to 96.1%. Correspondingly in the test cohort it showed accuracies between 80.0% and 91.7%. On classifying five molecular subtypes, the trained classifier with the MR radiomics phenotypes as sole source yielded an accuracy of 81.8%, which further reached a higher accuracy of 89.2% in the existence of histology diagnosis. Gutsche et al. implemented FET PET to enhance the diagnostic performance of their radiomics features on IDH genotype identification (34). The repeatability of the features was evaluated by calculating the intraclass correlation coefficient (ICC) and 297 features with robust discrimination ability were finally selected.

Deep convolutional neural networks (CNNs) and radiomics share the same procedure but have separate characteristics regarding radiological evaluation on IDH genotypes. Choi et al. reviewed 1166 preoperative CE-T1, T2, and FLAIR neuroimaging of gliomas grading II-IV derived from three centers and developed a CNN-based fully automated model hybridizing conventional MRI that integrated 2D tumor signal intensity and quantitative radiological features from 3D tumor shape and location, which was reproducible and generalizable for noninvasive characterization of IDH status in gliomas (35). Based on CE-T1, T2WI, and FLAIR from 1166 patients with gliomas (training cohort:727; internal test cohort: 129; external test cohort: 310), 20 out of 24 extracted features were selected and the classifier reached an AUC of 0.96, 0.94, and 0.86 respectively for training cohort, internal validation cohort, and external validation cohort.

The DNA-repairing enzyme O-6-methylguanine-DNA methyltransferase (MGMT) restores cytotoxic lesions in DNA caused by temozolomide chemotherapy, thus leading to drug resistance. Methylation epigenetically silenced *MGMT* has a connection with a better treatment response and a better prognosis than the unmethylated promoter (81). Based on a group of 105 patients with grade II-IV astrocytoma, Wei et al. established a radiomics model for *MGMT* promoter methylation prediction with supreme power (AUC: training cohort: 0.925, test cohort: 0.902), which successfully bisected the group into high-risk and low-risk subgroups for OS followed by temozolomide chemotherapy (39). *ATRX* mutation is another good prognostic factor that usually appears in LGGs accompanied by *IDH* mutation. Li et al. included 95 patients with LGGs and built a radiomics predictor of ATRX alterations, which was subsequently validated in an external cohort of 91 patients with good performance (AUC: training cohort: 0.94, internal test cohort: 0.925 and external test cohort: 0.725) (40).

Haubold et al. assessed the potential of multimodal imaging and radiomics algorithms to predict the grading and common mutations including MGMT of 42 patients with suspicious primary brain tumor (41). They applied 18F-FET PET along with MR Fingerprinting and extracted 19284 features from each patient, which were further divided into 32 for 1p19q codeletion, 64 for *IDH1*, 8 for *ATRX*, and 16 for *MGMT*. And through 5-fold cross-validation the AUCs in predicting the mutation were respectively assessed, with 1p19q for 97.8%, *IDH1* for 88.7%, *ATRX* for 85.1%, and *MGMT* for 75.7%. The 2016 WHO grading model yielded an AUC of 81.8% while AUC of discriminating LGG from HGG was 85.2%. Su et al. further investigated grading along with proliferation levels in 220 patients with various grades of gliomas (43). When combining radiomics features of multi-contrast MRI (T2WI fast-echo images (T2FSE), T1WI, FLAIR, CE-T1WI, DWI, ADC, PWI and CBF), the models displayed the highest AUC (0.911 for LGGs and HGGs, 0.896 for grades II–III, 0.997 for grades II–IV, 0.881 for grades III–IV, and 0.936 for levels of Ki-67 labeling index).

It has been reported that *CIC* mutation promotes glioma cell proliferation, differentiation, and aggression and results in a poor outcome (82–84). However, Zhang et al. found that patients with LGGs (*IDH* mutation) or oligodendroglioma (*IDH* mutation and 1p/19q codeletion) combined with *CIC* mutations may have better prognosis (42). As shown in MRI, LGGs (*IDH* mutation) with *CIC* mutation illustrate visually less malignant manifestations, such as fairer necrosis and more homogeneity among the tumor volume. They further developed a radiomics model to predict the *CIC* alterations based on 11 features derived from 120 patients with LGGs (AUC: 0.985).

Epidermal growth factor receptor (EGFR) variants are reported in 57% of GBM specimens. Among these, a deletion from exons 2–7, EGFRvIII is the most frequent EGFR variant, and extracellular domain (ECD) missense mutations like A289D/T/V, R108G/K, and G598V are the most frequent EGFR deletion comprising 10%–15% of transcription products while the deletion was found to co-occur with amplification (85). EGFR^A289V^ mutation has the most negative survival impact, which was reinforced by Binder’s study involving 260 patients with *de novo* GBM (38). To investigate the negative prognostic effect of EGFR^A289D/T/V^, Binder et al. firstly did quantitative imaging analysis comprising six different MRI modalities and extracted 2104 quantitative imaging phenomic features which were further reduced to a more manageable set of 299 using a multivariate classification framework (86). To promote radiographic interpretability, an experienced neuroradiologist further filtered these features into 17. The MRI signatures based on selected features presented a picture of decreased T1 signal but higher CE-T1 signal, higher T2 values, higher peak height (PH) values, and relative cerebral blood volume (rCBV) in EGFR^A289D/T/V^ mutant tumors region, indicating higher water content, hyperproliferation, and increased invasion in tumor region with EGFR^A289V^. The peritumoral edema region presented reduced fractional anisotropy (FA) generated by Diffusion Tensor Imaging (DTI) for cases with EGFR^A289D/T/V^ mutations, suggesting decreased tissue organization and homogeneity. Taking MRI signature and following modified cell lines *in vivo*, they demonstrated higher proliferation, increased aggressiveness, and shorter OS in EGFR^A289D/T/V^. To explore the mechanism *in vivo*, they inhibited two main signaling pathways of EGFR, RAS/RAK/ERK and PIK3CA/AKT, which revealed that A289V-induced EGFR activation mediates phosphorylated ERK and augments MMP1 expression which cause hyperproliferation and invasion. Finally, mAb806 targeting therapy was examined in EGFR^A289V^ mice models and was proven to be a potential therapeutic option as the mAb806 antibody reduced the tumor burden, inhabited tumor growth, and improved animal survival.

Glioma cells connect their microenvironment in a two-way street, mainly through cytokines and matrix proteins. POSTN is a secretory extracellular matrix protein made up of gliomas cells. Previous studies showed POSTN plays a role in neovascularization, endothelial junction formation decrease, stem cell maintenance, and macrophage recruitment (87, 88). Subsequent studies revealed POSTN in glioma grade, recurrence, and resistance to bevacizumab monoclonal antibody against VEGF-A (89, 90). Zinn et al. found GBM patients with different POSTN expression levels in association with distinct imaging features, which can be utilized in radiomics for prediction (32). They extracted 2480 radiomics features respectively in GBM patients and GSC-derived orthotopic tumors mice and selected 48 and 17 features respectively to build two classifiers (GBM AUC: 76.56%; mice, AUC: 92.26%).

F3T3 is a novel fusion proto-oncogene incorporating FGFR3-TACC3 found in approximately 3% of gliomas that functions as an important part in the activation of oxidative phosphorylation and mitochondrial metabolism. Though the foremost energy metabolism pathway of tumor is anaerobic glycolysis, GBM with F3T3 mutation depends on noncanonical mitochondrial pathway. Thus, F3T3 may serve as a potential target for targeting therapies such as mitochondrial inhibitors (91). Stefano et al. showed that, in the midst of IDH-WT tumors, F3T3-positive gliomas exhibit distinct molecular, radiological, and clinical features and possess a more optimistic clinical outcome independent of their grading. Their radiomics data composed 66 patients as training cohort and 78 patients from another institution as test cohort and identified F3T3-positive patients with good accuracy. They successfully built a classifier towards F3T3 mutation status (AUC: 0.87(training)/0.745(test)) and a model composing clinical, genetic, and radiomics profile to estimate the F3T3-positive patients’ OS as presenting the best concordance (C-index: 0.81). They further implemented multiple optimization techniques (SCCAN) to inspect the tropism of F3T3 gliomas for specific intracranial ROIs and finally located cortical and subcortical regions, especially insula and temporal lobe (36).

H3 K27M mutant occurs within the histone-3 gene (H3F3A) wherein an amino acid recurrently converts from lysine to methionine in the site 27, and H3 K27M-positive diffuse midline glioma is listed separately graded as IV in 2016 WHO classification (92). According to a report, H3 K27M-positive gliomas in thalamus area tend to result in a shorter median OS in pediatric patients than in H3 K27M-WT ones (93). Furthermore, there have been several epigenetic-targeted treatments towards H3K27M and an immunological study provided evidence for immunotherapeutic approaches like mutation-specific vaccines targeting H3K27M (94). Su et al. carried out a retrospective study on automated classification of H3 K27M genotypes. The Tree-based Pipeline Optimization Tool (TPOT), a method that automatically conducts feature and model selection procedure, along with pipeline optimization, was highlighted. The study included 40 H3 K27M-positive patients and 60 WT, of whom 75% were randomly grouped into the training cohort while 25% into test cohort. After extracting 99 features from FLAIR, TPOT finally refined 10 more manageable radiomics features and generated ten prediction models. The optimal model is generated through comparison of accuracy metrics. The model exhibiting the best performance in the test cohort yielded the highest average precision of 0.911 and AUC of 0.903, while validation in an independent validation dataset observed an average precision of 0.855 and an AUC of 0.85 (37).

Monitoring the core signaling pathway of GBM may reveal the tumor evolution, allow early clinical intervention, and enhance patients’ management (95). Park et al. built a radiogenomic classifier based on patients with IDH-WT GBM certified by next-generation sequencing (NGS), which noninvasively predicts retinoblastoma 1, p53, and Receptor tyrosine kinase (RTK) core signaling pathways (31). In this study, 85 patients were classified into the training cohorts in total and 35 into test cohorts, and their T1WI, T2WI, DWI, FLAIR, CE-T1WI, and perfusion MRI-like dynamic susceptibility contrast (DSC) were acquired for radiomics analysis. For each core signaling pathway, 71, 17, and 35 features passed extraction, and finally the top 5 features were selected respectively. Three models were evaluated, presented as AUC (RTK, training cohort: 0.87, test cohort: 0.88; p53, training cohort: 0.80, test cohort: 0.76; retinoblastoma, training cohort: 0.84, test cohort: 0.81). *PTEN* is a tumor suppressor gene participating in both ATK and RTK signaling pathways and the deficiency of *PTEN* is considered to be the main feature of GBM (96, 97). In addition to relying on sequencing and immunohistochemistry to detect *PTEN* alterations, Li et al. established a noninvasive radiomics method with good performance in guiding targeted therapy (AUC: training cohort: 0.925, test cohort: 0.787) (44).

### Differential Diagnosis

The radiological features of gliomas usually lack specificity, involving spherical well-encapsulated shape with ring enhancement indicating tumor angiogenesis and prominent peritumoral edema. The lesions are mostly multiple and are usually located at watershed or grey-white junction with white matter fiber bundles erosion. Further, the radiological features between malignant gliomas and lymphomas are analogous and there is also similarity in abscesses, infections, demyelinating diseases, and vascular lesions. The radiological features are also likely to be interfered with by hemorrhage, melanin, and paramagnetic ions.

Brain metastases (BMs) take up the second most common type of malignant brain neoplasms in adults preceded by GBM (98, 99). Early diagnosis is the key to appropriate therapies since the strategies for these two tumors are distinct with different local control rates and intervention prognosis: the prior treatment for GBM is maximum-safe surgery resection, following adjuvant radiotherapy and chemotherapy (100), while regarding BM the more effective and less invasive treatment is stereotactic radiosurgery (101). Qian et al. assessed high-dimensional radiomics features from T1-WI, T2-WI, and CE-T1 to distinguish GBM from solitary BM (47). In the retrospective study, patient’s population, including 242 GBM and 170 solitary BM, was randomly grouped (training cohort: 227, test cohorts: 185). An amount of 1303 radiomics features passed extraction, which were then refined by twelve feature selection methods. Thirteen classifiers were generated and all yielded excellent predictive efficacy with AUC≥0.95 in the training cohort. Through ROC curve analysis they found out that the combination of SVM and LASSO classifiers had the best prediction value in the test cohort (AUC: 0.90).

Primary central nervous system lymphoma (PCNSL) shares radiological similarities with GBM when solely using ADC parameter, due to some overlaps in ADC values. Kang et al. evaluated the feasibility of a radiomics model for the differentiation of atypical PCNSL and GBM based on ADC (45). The patient population in the training cohort contained 112 patients, while the population in the test cohort involved 42 patients for internal and 42 for external validation sets. They combined 12 feature selection methods with 8 classification methods using 5~50 selected features and optimized 8 ADC radiomics models. The prediction performance and stability were subsequently measured by each AUC and relative standard deviation (RSD) of each model. As a result, the ADC model combining RFE feature selection with RF classification yielded the highest diagnostic value with an AUC of 0.983 in the training cohort. The ADC model showed robustness exceling expert readers and was further assessed respectively in the internal validation cohort (AUC 0.984) and external validation cohort (AUC 0.944) to promote generalizability.

To standardize the procedure and improve efficacy, Wu et al. generated a novel radiomics system utilizing feature extraction and selection methods and classification framework based on dictionary learning and sparse representation (46). Simply using T2WI and CE-T1, they tested the technical feasibility of the system using 49 selected radiomics features out of 968 features extracted from 102 patients with PCNSL or GBM (training: test=67:35). The sparse representation radiomics system had superior PCNSL and GBM differentiation performance (training cohort: 98.51% accuracy, test cohort: 94.51% accuracy). Furthermore, the IDH1 prediction performance of the novel system exceeded traditional methods based on calculation by 11%.

### Prediction of Treatment Effect and Recurrence

Tumor recurrence in early posttreatment stage is commonly reported in HGGs. Assessing posttreatment MRI changes according to RANO standard within 0-72 hours is a common and effective method to evaluate the degree of surgery. Pseudoprogression (PsP) is a diagnostic dilemma presented as expanded and/or new regions of edema and enhancement, especially within the 2-5 months from the initiation of adjunctive therapies, which mimics tumor recurrence and radiation necrosis. The mechanism of PsP may be attributed to three factors: (1) non-tumor tissue chemoradiation damage, e.g., hemorrhage, ischemia, aseptic inflammation, edema and necrosis; (2) blood-brain barrier breakdown; or (3) other factors, e.g., signal artefacts from metal implants.

A recent retrospective radiomics study comprising 76 patients of histopathology-proved progressive disease (PD) and 22 of PsP from three centers by Elshafeey et al. provided evidence on perfusion MRI on accurately discriminating PD and PsP. Its reported model based solely on Ktrans maps had matching diagnostic value with the rCBV model in discriminating between PsP and PD. The final prediction model combining Ktrans with rCBV maps generated by SVM used the top 60 radiomics features ranking with MRMR, which achieved an accuracy of 90.82% and an AUC of 89.10% in discriminating between PsP and PD. Subsequent validation also showed statistical significance by LOOCV (AUC 89%) (50). Based on dynamic FET PET radiomics, Lohmann et al. aimed at establishing a reliable diagnostic test for differentiating PsP from tumor progression in gliomas patients (52). In the tumor segmentation process, data augmentation was implemented to increase the number of datasets from 34 patients to 102. The radiomics model was automatically generated using TPOT based on random forest classification, with an AUC of 0.74 in both training and test cohorts.

Kim et al. investigated the feasibility of multiparametric MRI radiomics incorporating diffusion and perfusion to identify tumor recurrence within 3 months following standard therapy (48). They developed and validated a radiomics model comprising CE-T1WI, FLAIR, ADC, and CBV maps using 61 patients as training cohort and 23 patients as validation cohort. Initially 6472 features were extracted and then 12 significant radiomics features passed selection using LASSO to construct the integrated model. And the model presented best diagnostic performance (AUC, 0.90) over any single imaging technique or parameter model. The internal validation (AUC, 0.96) and external validation (AUC, 0.85) cohorts strengthened the outcome.

Towards differentiating tumor recurrence from radiation necrosis, Wang et al. carried out a radiomics study involving 112 patients as training cohort and 48 patients as test cohort. The multidimensional quantitative model integrated clinical information (patients’ individual features and gliomas grade) and radiomics information (MRI techniques (T1WI, T2WI, CE-T1WI and FLAIR) and PET images using both 18F-FDG and 11C-MET), while the radiomics model only included radiomics information. Fifteen textural features were selected from the images for the construction of radiomics model and integrated model. And the integrated model showed significant superiority over radiomics model (both training and test cohorts: p < 0.001) and was proved to be accurate and effective in the prediction of differentiating postoperative tumor recurrence from radiation necrosis (training cohort: AUC 0.988, test cohort: AUC 0.914) (53).

Brain necrosis after radiotherapy is a common complication in approximately 3%–24% of patients (102), mostly with primary or metastatic cancer of the head, neck, and CNS. Bevacizumab has shown its potential in symptomatic relief and radiographic response compared with general corticosteroid therapy in randomized study (103), however, some patients are unable to gain benefit or even worsen. To predict the treatment effect of bevacizumab in brain necrosis patients, Cai et al. developed a radiomics model based on a total of 149 patients including 42 as an external validation cohort, which yielded an AUC of 0.916 in the training cohort and 0.912 and 0.827 in the validation cohorts (51).

Immune checkpoint inhibitors (ICI) hold great promise for GBM treatment, however the suppressive microenvironment of GBM characterized by poor antigen presentation and low T-cell activation and infiltration limits the ICI application. Aslan et al. investigated mechanisms of resistance to ICIs blocking PD-1 and CTLA-4 and acquired immune heterogeneity in the allogeneic intracranial inoculated mice with Gl261 tumor cells (49). To determine the response of ICI in mice post inoculation and identity PsP, they built a radiomics signature based on CE-T1WI and T2-WI. From 101 mice inoculated with Gl261 tumor cell before and during ICI treatment, they extracted 423 features and built a gradient boosting classifier containing all 423 z-score-normalized radiomics features with an accuracy of 82.7%. Subsequent *in-vivo* and *ex-vivo* experiments proofed that PD-L1/PD-1/CD80 axis plays an important role in ICI resistance induced by CD4 T cell suppression, tumor-associated macrophages, and Treg extension in the microenvironment of GBM.

### Predicting Patient’s OS and Complications

According to a large-scale randomized trial, the median survival time of GBM is 14-15 months, which can be prolonged by adjuvant temozolomide with radiotherapy (104). However, the current radiotherapy plans ignore the biological heterogeneity of individuals and use the same dose, resulting in significant difference in patients’ OS (105). Radiomics and radiogenomics can provide an imaging biomarker on predicting the individual radiotherapeutic response, which helps to adjust the dose and make a personalized treatment plan. By combining the clinical risk factors and radiomics signature which was built with 152 patients with GBM to predict the radiotherapeutic response, Pan et al. developed a nomogram to predict the OS, with C-indexes up to 0.764 and 0.758 respectively in the training cohort and external validation cohort (54).

The study by Dastmalchian et al., including 31 patients with GBM, LGGs, and metastases and 20 top selected features, proved that the radiomics approach has robust potentiality to differentiate between these tumors and to predict OS of GBM (56). They found a significant difference between patients with different selected features such as T1/T2 entropy and secondary features like high-gray run emphasis (p <0.05). And the cut-off values dichotomizing the GBM patients’ median survival were calculated by grading these features. For example, lower entropy values in solid tumor regions (p:  0.034) in T2 maps correlate with longer survival of 11 months and 6.7 months for those below the cut-off value, and higher entropy values in peritumoral white matter regions (p:  0.009) in T1 maps correlate with longer survival of 18 months and 6.8 months for those below the cut-off value.

Since the mutation of IDH1-R132H and MGMT in GBM patients is strongly associated with the OS and PFS, Bae integrated radiomics with clinical and genetic profiles and built several models to predict the prognosis of 217 GBM patients (training: test=3:1) (59). From 796 features derived in T1WI, T2WI, FLAIR, postcontrast 3D T1WI, and DTI, they selected 18 significant features and trained multilayers RSF models. Except for the integrated model, the model containing only radiomics features was the most significant with successful validation in the test cohort (OS: iAUC 0.652, PFS: iAUC 0.590).

Papp et al. evaluated the prediction value on dichotomized OS using an integrated model comprising 56 features including 11C-MET PET radiomics characteristics *in vivo*, histopathological characteristics *ex vivo*, along with patients’ individual information to predict survival in glioma patients without treatment (58). The cut-off value determines 36 months as the survival prediction threshold and the prediction weight for each model was assessed in training cohorts and the validation cohorts. When it came to validation, they introduced the Monte Carlo cross-validation (MCCV) different from k-fold validation in that the sample may appear multiple times in the same set (training set/test set). The MCCV proved the highest AUC for the integrated model as 0.9, following the patient-based and histopathology-based models.

It has been reported that in GBM patients, females exhibit longer OS compared to males, which may be associated with hormonal, metabolic, and immune variances. Based on the discovery, Beig et al. developed sexually dimorphic radiomics risk score (RRS) models to predict patients’ OS. The OS prediction model combines age molecular features, extent of resection, and RRS, showing good performance in both male and female cohorts [0.73/0.88(C-index, male), 0.73/0.69(C-index, female)]. By further analyzing radiogenomics associations between MRI-based phenotypes and transcriptomic data correspondingly, they found that RRS is associated with a series of biological activities including angiogenesis, apoptosis, cell differentiation, cell proliferation, and cell adhesion (60).

Verduin et al. involved the training (n = 142) and validation cohort (n = 46) to establish a combined model for prognosis of OS in IDH-WT GBM patients based on quantitative radiological features, qualitative Visually Accessible Rembrandt Images (VASARI) features, and clinical information. The accuracy and reproducibility of the combined model was analyzed using Harrell’s C-index (training cohort: 0.72, validation cohort: 0.73). They additionally developed a prediction model towards molecular mutation status comprising IDH, MGMT methylation, and EGFR amplification in 95 patients for the training cohort and 38 patients for the validation cohort. In this model, 5 VASARI and 5 radiomics features mainly selected from T2WI were considered to be most prognostically relevant, with performance towards MGMT methylation (AUC: 0.667) and EGFR amplification (AUC: 0.707) yielding significance in external validation cohorts (61).

A radiomics study on PFS and OS stratification by Kickingereder et al. included 181 GBM patients available of imaging information (CE-T1, FLAIR, and T2WI), DNA methylation profiling (MGMT methylation status and global DNA methylation pattern), treatment (surgery, TMZ chemotherapy and/or radiation), and patients’ individual information (62). In the prognostic analysis, a total of 386 features were selected independently in a test-retest MRI cohort. Subsequently, 8 of these were further used to construct a radiomics signature using sole radiomics information. When using only epigenetic and clinical information, the prediction error for PFS (29%) and 37% for OS (27%) is not appreciable, which were reduced by 36% for a model after integrating radiomics signature. The radiomics signature showed significance beyond models using other information (P ≤ 0.01).

The epilepsy complicated by LGGs is mainly attributed to compression and stimulation of the brain tumors that cause the degeneration and gliosis of the brain cells around the tumors which constitute the epileptic foci complex. Wang Y aimed at predicting epilepsy types to guide more targeted antiepileptic therapy in a retrospective study. A novel radiomics nomogram was developed with 4 selected discriminative MRI features regarding location and molecular background in 205 LGG patients, which displayed excellent quantitative clinical prediction performance (AUC: 0.863) (106). Qian et al. suggested a radiomics risk score to alternatively identify the OS in LGGs. When combined with independent clinical prognostic parameters such as WHO grade, age at diagnosis, and seizure, the nomogram based on the risk score exhibited high prognostic accuracy (C-index: training cohort: 0.92, test cohort: 0.70) (57). They subsequently implemented radiogenomic analysis of high-risk positively associated genes, further revealing the underlying correlated biological processes including hypoxia, angiogenesis, and apoptosis. For the prediction of PFS, Liu et al. worked out a practical nomogram incorporating clinicopathologic factors and a radiomics signature based on 300 patients with LGGs (C-index, training cohort: 0.684; validation cohort: 0.823) (55), and demonstrated similar biological processes through radiogenomic analysis.

## Brain Metastases

Approximately 20% of the cancer patients with other primary sites develop brain metastases, outnumbering primary brain tumors 10:1, but the actual statistic is estimated to be even more since plenty of them do not go through regular MRI examination. The top three extracranial primary cancer types with high intracranial metastatic tendency are lung cancer, breast cancer, and melanoma, which respectively have incidences up to 20-56%, 5-20%, and 7-16% (107–110). Meanwhile, the incidence of brain metastases’ occurrence after primary cancer varies according to race, age, and primary cancer. Brain metastases’ main symptom is parallel to space occupying lesions, which varies with the lesion location.

### Evaluation of Mutation Status

Since there is inter-heterogeneity between the primary tumor and metastases, assessing the mutation status in the metastases region and comparing with the primary tumor are meaningful in guiding individualized treatment. EGFR inhibitors, such as erlotinib and gefitinib, received distinct responses in GBM and NSCLCs patients originated from different EGFR mutation sites. In NSCLCs, the mutation sites typically locate in the kinase domain that facilitate sensitivity to first-generation EGFR inhibitors. Unlike NSCLCs, the mutation sites of GBMs locate in extracellular domain that promote resistance (111).

Ahn used CE-T1 MRI to predict the EGFR mutation in histologically certified primary lung cancer patients’ brain metastases (33 with EGFR WT, 29 with EGFR mutation) (112). Among all the combination of 7 feature selection methods and 4 classification methods, the RF classification model applying RF selection yielded highest AUC of 86.81% on predicting EGFR mutation status. Subsequent analyses subgrouping BMs by measurable size revealed smaller BMs correlate with better discrimination capacity (AUC 89.09% in the small BMs subgroup, combining SVM classification with RF selection).

Park used DTI and T1-contrast to classify the EGFR mutation in 99 BMs from 51 NSCLC patients, verified by biopsy. Among all the combinations of 5 feature selection methods and 4 classification methods, the linear discriminant algorithm classifier using 5 features selected by tree-based methods showed the best diagnostic performance, resulting in an AUC of 0.73 (113).

Chen did a retrospective study using CE-T1, T2WI, and FLAIR to predict the mutation on EGFR, ALK, and KRAS mutation in BMs from patients diagnosed primary lung cancer, verified by genotype testing. The model on EGFR, ALK, and KRAS incorporating both radiomics and clinical information resulted in AUC values of 0.912, 0.915, and 0.985 (114).

### Identifying Primary Tumor

The clinical manifestations of BMs are analogous to primary brain tumors. Generally, systemic metastases, cachexia, and multiple foci in CNS may indicate BMs, however, there are up to 15% with unknown primary tumor (115). Kniep retrospectively studied 189 patients with primary breast cancer, lung cancer (NSCLC and SCLC), gastric cancer, or melanoma, who developed BMs, and analyzed CE-T1 and nonenhanced T1WI and FLAIR through machine learning algorithm (29). The results showed that all the RF classifiers surpassed senior neuroradiologists’ reading. After combing radiomics and clinical data, the 5-class model showed best prediction performance with lowest AUC (0.64) for NSCLC and highest AUC (0.82) for melanoma.

### Prediction of Recurrence

As noninvasive treatments such as radiation and chemotherapy have more extensive application in BMs, the most common application of radiomics in BM in recent years may be prediction of treatment and progression.

Prasanna proposed a novel entropy feature called co-occurrence of local anisotropic gradient orientations (COLLAGE) which is of great prognostic value in evaluating radiation necrosis and tumor recurrence on gadolinium-contrast T1WI (116). They proved in 75 patients with metastatic brain tumors that, with additional independent multisite validation, COLLAGE features exhibited statistically significant different skewness (P <0.05) in recurrent tumor patients compared to patients with pure tumor and cerebral radiation necrosis > 80%.

Huang retrospectively analyzed 161 patients with NSCLC (576 brain metastases) postoperatively by Gamma Knife radiosurgery and found zone percentage related to progression (117). After feature selection by consensus clustering, analysis of univariate Cox proportional hazards model comprising clinical variables, and radiomics features revealed potential prognostic factors that were subsequently selected to build a multivariate Cox proportional hazards model, which indicated that a textural feature called higher zone percentage was independently pertained with higher local tumor control rates (HR 0.712; P = 0.022). Similar to the result, multivariate proportional hazards model in cause-specific condition also filtered higher zone percentage (HR 0.699; P = 0.014).

To predict diagnosing treatment effect after stereotactic radiosurgery, Peng investigated 82 lesions of BM with obvious progression from 66 patients who underwent SRS based on CE-T1 and T2WI MRI (30). Five top-performing radiomics features out of 51 extracted features filtered by univariate logistic regression were selected to build a subsequent hybrid IsoSVM model, which was assessed by the LOOCV (AUC 0.79). Mouraviev retrospectively analyzed 408 BM lesion in 87 patients who underwent SRS based on their pretreatment CE-T1, T2WI, and FLAIR (28). For 440 extracted radiomics features, they applied RF feature importance and ranked these features for selection. The top 12 features comprising radiomics and clinical data are optimized for best prediction model, with the highest AUC (mean = 0.793).

## Limitations

Radiomics is a rapidly expanding field and is still in extensive clinical exploration stage, with many obstacles to overcome. We may discuss the limitations from the aspects of standardization, robustness, repeatability, reproducibility, and generalizability.

Standardization is the basis of robustness, producibility, and generalizability. Current standards lack results validation, incomplete results reports, and unidentified confounding variables in the source database, especially for retrospective data. To solve the above problems and standardize radiomics-specific reporting, Lambin et al. put forward an evaluation system comprising 16 weighted metrics to determine the workflow completeness, model quality, and clinical adaptation potentiality of radiomics studies, in the form of the radiomics quality score (RQS) (12). The establishment of RQS extended a number of initiatives, such as the Transparent Reporting of a multivariable prediction model for Individual Prognosis OR Diagnosis (TRIPOD) consensus (118).

As is discussed in the Radiogenomics section, radiomics and radiogenomics can only identify the correlation, thus lacking robustness and credibility without tissue biopsy. As for radiomics itself, the accurate segmentation of ROIs is the most challenging step that largely affects the robustness of outcome. Due to a tumor’s heterogeneity and polymorphism, manual segmentation is used in most imaging studies. Its advantages are high accuracy and fine delineation of irregular tumor boundaries, but it is greatly affected by subjective factors and is time-consuming and inefficient, with low repeatability. Recently, novel volume data segmentation methods based on deep learning models, such as CNNs named after the shape of the feature map structure (U-net (119) V-net (120), W-net, UNet++ (121) and Y-net), and DeepMedic have made a breakthrough in the clinical radiology segmentation. Current studies demonstrated the utility of CAD, which combines automated brain tumor segmentation with radiomics, in helping physicians to detect following initial observation (122, 123). Most normal tissues like bones and organs can be segmented semi-automatedly or fully automatedly.

However, current protocols of autosegmentation approaches are diverse and lack unified standards. From the studies we reviewed, more intelligent algorithms such as deep learning are rarely used in radiomics of brain tumor compared to lung cancer, prostate cancer, and colorectal cancer. Current segmentation, feature selection, and classification methods in brain tumors are mainly manual operations using shallow machine learning methods, such as random forest, SVM, and LASSO. What’s more, the ratio of articles with test-retest analysis is low for currently available original research, which also adds doubts upon repeatability and reproducibility of radiomics analysis. Though the sensitivity, specificity, and/or AUC of the reviewed studies are considerable, few are prospective studies that were later followed up or confirmed by biopsy.

Another problem is that the clinical translation of radiomics studies in multicenter studies faces difficulty in repeatability and reproducibility. For example, MR images may capture noise caused by physiological motion, magnetic field, eddy current, and unsteadiness of the scanning hardware. Then during image reconstruction, noise is post-processed to be wiped out prior to ROIs determination (124). As MR images omit physical parameters, such as magnetic field strength and voxel size, the various settings of image acquisition, reconstruction algorithm, and image processing makes MR radiomics more challenging than CT to ensure repeatability and reproducibility (125–127).

To test the generalizability of prediction model, there are the internal validation and external validation using other centers’ data. Current studies in brain tumor still lack big populations, especially from multiple centers. A systematic review by Park et al. evaluated 51 original radiomics research articles in neuro-oncology with RQS and showed that only 29.4% performed external validation, with few studies discussing clinical utility and none of them conducting phantom study or cost-effectiveness analysis (128).

There are also ethic problems in that, while the development of AI algorithms requires not only fundamental techniques, but also legislation and perhaps ethics, there are issues on whether researchers or governments should be motivated to share private validated data for machine learning (129). What’s more, it is possible for AI algorithms to be tampered with improper intention to make profits.

## Further Developments in Brain Tumor

Radiomics accelerates the development of precision medicine. In addition to providing accurate and well-organized personal radiological diagnostic information to identify different states for each patient, large quantities of features extracted from numerous pathologically confirmed patients can contribute a lot to a large-scaled database for tumor classification. The Picture Archiving and Communication System (PACS) has enabled the acquisition, display, processing, storage, transmission, and management of medical images to be digitized and networked in a uniform standard (130, 131), with parallel progress in Europe (132) and developing countries (133, 134). Additionally, there are strong public repositories which record these systematic electronic radiological data with open access, for instance, The Cancer Genome Atlas (TCGA) (135), The Cancer Imaging Archive (TCIA) (136), and The Quantitative Imaging Network (QIN) (137).

As the database improves, it can be used for deep learning to evolve over time to build more sophisticated classifiers and may help discover more internal connections between image features and gene expression. Open availability of source code and data is encouraged for current radiomics studies to promote technical development. The development of fully automated approaches based on deep learning will start from solving the most common clinical problems with plenty of data (138). These clinical problems may concern occasions where professional neuroradiologists are in heavy demand or analyzation is too cumbersome for neuroradiologists, like predicting *IDH* mutation status in gliomas (35).

The development of radiomics also compels the development of histopathology. Sampling is a crucial step in identifying the tumor, but it relies on the location of the lesion and is often affected by operator’s subjective factors and intra-heterogeneity and inter-heterogeneity of tumor. If radiomics is performed before sampling, it can segment the lesion and suggest the most interesting area so that we can puncture the target tissue accurately. Multicenter radiomic research requires establishing norms for the radiomics study protocols and for their reporting in the literature, which also supplements traditional imaging reports with quantitative indicators and more standard structures.

Based on multiple noninvasive biomarkers, more explicit characteristics of tumor can be assessed, and the progression of tumor can be recorded and visualized (139). For example, liquid biopsy enables the analysis of molecules or macrostructures in low concentration from body liquid that shows minimal invasiveness towards patients who are susceptible to tumor or cannot withstand biopsy. Cucchiara et al. integrated liquid biopsy and radiomics to monitor clonal heterogeneity of *EGFR*-Positive NSCLC (140). As a result, more individualized treatment plans can be tailored and patients with imperceptible premalignant lesions or who undergo surgery can also benefit, though the expense is another problem to be discussed. Future studies should focus on improving the sensitivity and specificity.

## Conclusion

Radiomics was born from traditional radiology, bioinformatics, and machine learning and provides clinicians with economical, automatic, and accurate diagnosis on brain tumors by mining high-dimensional data correlated with lesions extracted from images. The overall imaging and evaluating by radiomics not only presents the inner heterogeneity of the lesion but also indicates the microenvironment surrounding the tumor region, making it possible to guide targeted agents before experiment or to be aligned with biopsy to maximize the clinical implications. Though many guidelines are published or being developed, there are still gaps in standard radiomics reporting. As more sophisticated segmentation and analyzation techniques are exploited, along with big data to reach multicenter interoperability, we believe radiomics will soon expand rapidly beyond a small research area and transform into a clinical surrogate diagnosis tool.

## Author Contributions

ZY and YZ reviewed the literature and wrote the manuscript. LL reviewed the literature and generated **Table 2**. YZ and ZL designed the research. All authors contributed to the article and approved the submitted version.

## Funding

The research was funded by China Postdoctoral Science Foundation 2018M643006.

## Conflict of Interest

The authors declare that the research was conducted in the absence of any commercial or financial relationships that could be construed as a potential conflict of interest.

## Publisher’s Note

All claims expressed in this article are solely those of the authors and do not necessarily represent those of their affiliated organizations, or those of the publisher, the editors and the reviewers. Any product that may be evaluated in this article, or claim that may be made by its manufacturer, is not guaranteed or endorsed by the publisher.

## Abbreviations

AUC, area under the curve; BM, brain metastases; CAD, computer-aided diagnosis and detection; CADe, computer-aided detection; CBV, cerebral blood volume; CCC, concordance correlation coefficient; CE-T1WI, contrast-enhanced T1-weighted MRI; CNN, convolutional neural network; DSC, dynamic susceptibility contrast; DTI, diffusion tensor imaging; DWI, diffusion-weighted imaging; EGFR, epidermal growth factor receptor; FLAIR, fluid-attenuated inversion recovery; GBM, glioblastoma; HGG, high-grade glioma; IDH, isocitrate dehydrogenase; LASSO, least absolute shrinkage and selection operator; LGG, low-grade glioma; MGMT, O-6-methylguanine-DNA methyltransferase; MRI, magnetic resonance imaging; mRMR, minimum redundancy maximum relevance algorithm; MRS, magnetic resonance spectroscopy; NFPA, non-functioning pituitary adenoma; OS, overall survival; PA, pituitary adenoma; PCA, principal component analysis; PCNSL, primary central nervous system lymphoma; PD, progressive disease; PFS, progression-free survival; PsP, Pseudoprogression; PWI, perfusion-weighted imaging; RECIST, Response Evaluation Criteria in Solid Tumours; RF, random forest; RFE, recursive feature elimination; ROC, receiver operating characteristic curve; RQS, radiomics quality score; SAM, significance analysis of microarrays; SVM, support vector machine; TRC, treatment-related changes.
